# Validation of canine prostate volumetric measurements in computed tomography determined by the slice addition technique using the Amira program

**DOI:** 10.1186/s12917-019-1778-z

**Published:** 2019-02-04

**Authors:** Katharina Haverkamp, Lisa Katharina Harder, Nora Sophie Marita Kuhnt, Matthias Lüpke, Ingo Nolte, Patrick Wefstaedt

**Affiliations:** 1 Small Animal Clinic, University of Veterinary Medicine Hannover, Foundation, Hannover, Bünteweg 9, D-30559 Hannover, Germany; 2 Institute for General Radiology and Medical Physics, University of Veterinary Medicine Hannover, Foundation, Hannover, Bischofsholer Damm 15, House 102, D-30173 Hannover, Germany

**Keywords:** Prostate gland, Computed tomography, Dog, Volume, Amira

## Abstract

**Background:**

Prostatic diseases are common and mostly associated with enlargement of the accessory gland. Thus, determining the prostate size has become a main criterion for evaluating prostate health status. Computed tomography (CT) is recommended as a beneficial tool for evaluating prostate size, morphology and surrounding tissues. The purpose of this study was to establish an accurate procedure for volume estimation and afterwards evaluate the prostate volume in CT. Data of 95 dogs were analysed (58 male intact, 37 male neutered) using the slice addition technique with the Amira program. Accuracy of volumetric measurements by CT was validated by comparing them with those of phantoms of known volume. Patients were grouped according to age (< 4 yrs., 4–8 yrs., > 8 yrs) and prostate morphology in CT (H = homogeneous, I = inhomogeneous, C = cystic). The length of the sixth lumbar vertebra was measured to relate prostate volume to body size. This ratio was generated to compare prostate volume between the groups, irrespective of body size (ratio volume = Rv).

**Results:**

A high correlation between the CT-derived and phantom volume was found. Overall, the mean prostate volume was 58.6 cm^3^. The mean ratio volume was 1.3 in intact male dogs, this being significantly higher than in neutered dogs (0.7). The lowest ratio volume values were found in group H for intact (Rv = 0.9) and neutered dogs (Rv = 0.6), followed by group I (intact: Rv = 1.1; neutered: Rv = 0.7) and C (intact: Rv = 1.4; neutered: Rv = 0.8). The length of the sixth lumbar vertebra was well correlated with the prostate volume (intact: r = 0.63, *p* < 0.001; neutered: r = 0.48, *p* = 0.003), while age exhibited a correlation only in intact dogs (r = 0.52, *p* < 0.001).

**Conclusion:**

The present study is pioneering in applying a slice addition technique to volumetric measurements of the prostate gland in CT, resulting in a highly precise method. Volumetric measurements of the canine prostate gland in CT images provide information about the prostate structure, castration status, age and body size of the patients. Therefore, prostate volume is a relevant parameter for evaluating prostate health status.

**Electronic supplementary material:**

The online version of this article (10.1186/s12917-019-1778-z) contains supplementary material, which is available to authorized users.

## Background

Prostatic diseases including benign prostatic hyperplasia (BPH), prostatitis, abscesses and cysts are common, especially in older dogs [1]. Symptoms associated with prostatic diseases are similar and non-specific [2–4] like fluid dripping, haematuria, strangury, staining to defaecate, pain on rectal or abdominal palpation and deficits of hind limbs [1, 3, 5]. As most disorders involve prostate enlargement, examining the size has become an important tool for evaluating prostate health status [6]. The prostatic dimensions have been evaluated in several studies in x-rays, ultrasound and computed tomography (CT) images [7–19]. The CT is recommended as a reliable tool for investigating prostate size and tissue as well as surrounding structures by several studies [16–18]. Choi et al. reported that prostate boundaries were more readily visible with CT than with ultrasonography [19] and Kuhnt et al. described that prostate lesions are more visible with CT than with ultrasound [16]. To minimise the effects of a patient’s body size, the ratio between prostate dimensions and the length of the sixth lumbar vertebra was calculated for comparing results [16–19]. Different studies recommended the prostate width and length as useful parameters to describe prostate size in CT [17, 19]. Other authors recommended prostate width and height instead [18]. Thus, there seems to be no uniform enlargement pattern of the canine prostate gland, which was also proven by Atalan et al. [11]. They showed that canine prostate enlargement was stronger in length and height than in width in ultrasound measurements. Therefore, measuring one single dimensional parameter might lead to misinterpretation of prostate size. For this reason, volumetric measurements, combining single dimensional parameters like height, length and width, might be more suitable for determining prostate size and an advantage in diagnosing prostate enlargement.

Schulze et al. measured prostate volume in CT with a formula of an ellipsoid body but accuracy was not verified [20]. However, previous studies investigated prostate volume by means of different formulas in ultrasound [9–14, 21] (Table 1), resulting in over- or underestimation of the actual prostate volume measured by water displacement [13, 14].

**Table 1:** Formulas used to calculate the prostate volume of dogs in different studies

Authors	Formula
Nair et al. [15]; Atalan et al. [10]	V = 0.487 x L x W x (DL + DT)/2 + 6.38
Ruel et al. [12]	V = L x W x He × 0.523
Kamolpatana et al. [13]; Ghadiri et al. [14]	V = L x W x D × 0.524
Kamolpatana et al. [13]	V = L x W x D
Ghadiri et al. [14]	V = 0.584 x L x L x He
Kamolpatana et al. [13]	V = {1/2.6 (L x W x D)} + 1.8

The measurements were performed in ultrasound

*V* Volume, *He* Height, *L* Length, *W* Width, *D* Depth, *DL* Depth longitudinal, *DT* Depth transversal

Lee et al. and Choi et al. used a volume-rendering software tool to measure the prostate volume in CT data. The actual prostate volume was unknown in these studies [17, 19].

The slice addition technique is recommended for measuring the volume of different organs like canine kidney, liver and spleen [22]. Nonetheless, verification regarding accuracy in measurements of the canine prostate gland is missing. Thus, further studies are needed to investigate the accuracy and diagnostic benefit of CT-based prostate volumetric measurements, especially for different ages, breeds and dogs with prostatic disorders.

The aim of the present study is to measure the canine prostate volume using a slice addition technique. The accuracy of this technique shall be verified by the usage of phantoms with known volume as well as by volumetric measurements of canine prostates ex vivo. Furthermore, the volume of the prostate gland in dogs shall be analysed with the validated technique regarding differences due to age, body size and prostate structure. In past literature, the length of the sixth lumbar vertebra was used to relate prostate dimensions to the body size of the examined dogs [16–19], but correlation of prostate size with the length of the sixth lumbar vertebra was not checked. Therefore, the final aim of this study is to investigate whether and to which extent prostate volumes correlate with the length of the sixth lumbar vertebra.

## Methods

### Patients

In this retrospective study, CT data sets of dogs that were presented to the Small Animal Clinic, University of Veterinary Medicine Hannover, Foundation between October 2007 and August 2017 were reviewed. The following criteria were established for patients to be included in this study: male intact dogs aged eight months or older were included if abdominal CT examination data sets with and without contrast agent and no imaging artifacts such as high density streaks caused by metal implants existed. The scans should include the whole prostate as well as the sixth lumbar vertebra to put the prostate size in relation to body size. Dogs castrated by hormone substitution were excluded. Ninety-five patients met the criteria. The mean age was 7.6 years and mean body weight 28.4 kg. Fifty-eight male intact (mean age 7.3 years/mean weight 30.8 kg) and 37 neutered male dogs (mean age 8.2 years/mean weight 25.5 kg) were included in this study (Additional file 1).

### CT data acquisition

All scans were performed with a 64-multi-detector-row CT scanner (Phillips Brilliance 64, Philips GmbH, Hamburg, Germany). Abdominal CT scans were performed in dorsal or ventral recumbency with a voltage of 120 kV, slice thickness of 2 mm, pixel sizes ranging from 0.15 × 0.15 mm to 0.84 × 0.84 mm and a pitch of 1.171. Depending on the patient’s body symmetry change, an automatic current selection function (DoseRight-D) modulated current during tube rotation, which resulted in different mAs-products between the dogs. Patients were anaesthetised with levomethadon (L-Polamivet 0.2 mg/kg; CP-Pharma Handelsgesellschaft mbH, Burgdorf, Germany), diazepam (Ziapam®, 0.5 mg/kg, Laboratoire TVM, Lempdes, France) and propofol (individual dose depended on effect; Narcofol® CP-Pharma Handelsgesellschaft mbH, Burgdorf, Germany). Anaesthesia was maintained by inhalation with isoflurane (Isofluran CP®, CP-Pharma Handelsgesellschaft mbH, Burgdorf, Germany) during CT examination. First, a native scan was performed. A second scan was carried out after a non-ionic iodinated contrast agent (Xenetix® 300, Guerbet GmbH; Sulzbach, Germany) had been injected into the vena cephalica antebrachii or vena saphena lateralis via a power injector (MedRad Vistron CT® 610 System, Indianola USA, 2 mL/kg; flow rate: max. 3 mL/sec; duration: max. 30 s). The automatic scan in the prostate region of interest (ROI) was started 49 s after a threshold of 150 Hounsfield units in the aorta had been exceeded, which was tracked by a local ROI positioned in the aorta in transversal view. Afterwards, CT data sets were stored in DICOM format.

### CT image analysis and volumetric measurements

**Fig. 1:**
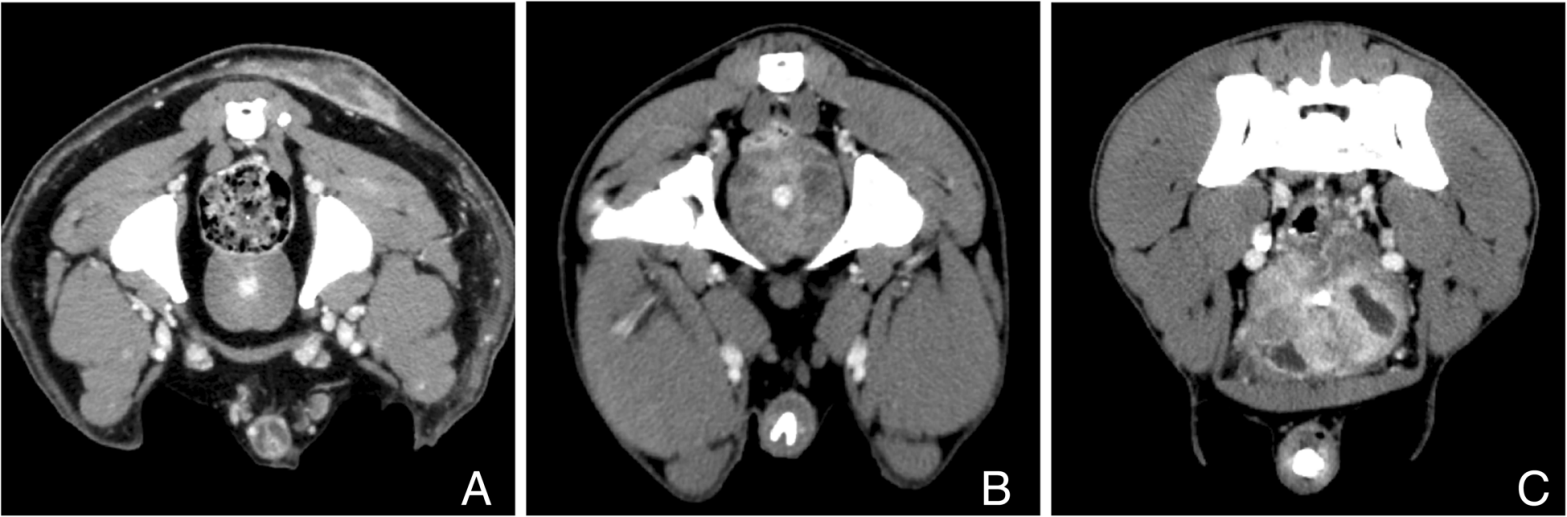
Different prostate structures in CT images. **a** homogeneous prostate, **b** inhomogeneous prostate, **c** cystic prostate

Morphological analysis of the prostates was carried out with an image-processing workstation (Extended Brilliance Workspace, Philips Medical Systems, Ohio, USA). Each prostate was analysed by the same observer (first author) to avoid inter-observer variability. The prostates of male intact and neutered dogs were assigned to one of three groups depending on the morphological appearance of the prostate in CT imaging: prostates with homogenous tissue structure (H), prostates with inhomogeneous tissue structure (I) and prostates with cystic tissue structure (C; diameter of cysts ≥1.2 mm) (Fig. 1) [16].

Additionally, the patients were categorised into three groups depending on their age: younger than four years (group 1), four-eight years (group 2) and older than eight years (group 3) [16].

The allocation to age groups and structure groups are shown in Table 2.

**Table 2:** Patient’s allocation to age groups and structure groups

		Intact Male Dogs(58)				Castrated Male Dogs (37)			
		Total	H	I	C	Total	H	I	C
	Total	58	6	9	43	37	20	10	7
< 4 yr	18	12	6	2	4	6	4	2	0
4–8 yr	30	19	0	6	13	11	5	3	3
> 8 yr	47	27	0	1	26	20	11	5	4

*H* homogeneous, *I* inhomogeneous, *C* cystic

**Fig. 2:**
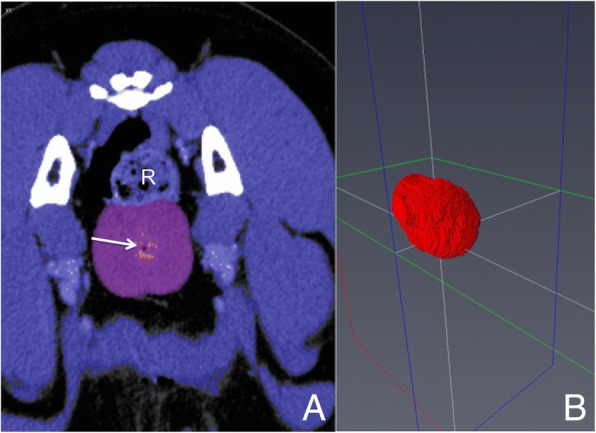
Assessment of canine prostate volume with Amira using the slice addition technique. Image **a** For volume analysis, the prostate gland was marked (purple) and can be located ventral of the rectum (R) as a bilobed structure. The urethra is included in the encircled area (arrow). Image **b** shows a 3-D reconstruction of the segmented prostate using Amira software

The prostate volume was determined by a specific software (Amira 6.2; FEI, part of Thermo Fisher Scientific, Hillsboro, Oregon, USA). In order to determine the prostate volume by means of Amira, it was necessary to segment the gland from the surrounding tissue. Due to low contrast between the prostate and surrounding structures, no automatic segmentation was feasible and manual segmentation was performed in scans with contrast agent. Therefore, the prostate gland was encircled manually with the mouse curser tool in transversal image view within all slices. The urethra (Fig. 2, arrow) was not excluded from the measurements. To compute the total prostate volume, the number of voxels within the labelled area multiplied by the size of a single voxel were added up for each marked slice.

The length of the sixth lumbar vertebra was measured in sagittal view. Therefore, a horizontal line was drawn towards the middle of the corpus vertebralis parallel to the vertebral canal. To relate prostate volume to body size, the ratio between the cubic root of the volume to the length of the sixth lumbar vertebra was determined. Hereafter, this ratio is referred to as “ratio volume” (Rv).

### Phantom and cadaver measurements

**Fig. 3:**
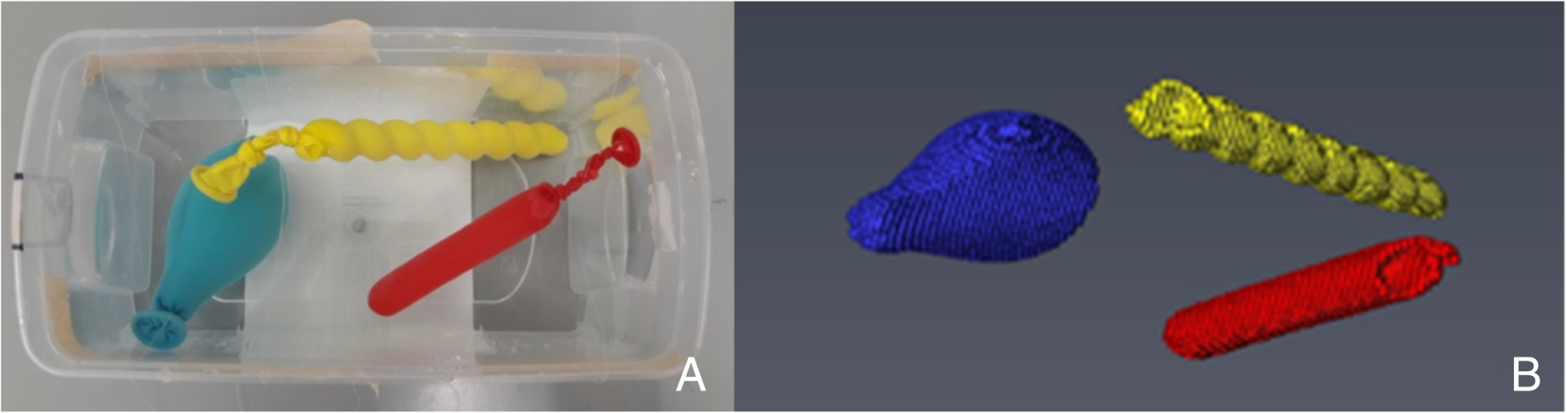
Phantom measurements. Image **a** shows differently shaped balloons filled with a known volume of water and contrast agent. For scanning, phantoms were positioned in a water-filled bucket. Image **b** shows reconstruction of segmented phantoms using Amira software

To evaluate the accuracy of the CT-based volume measurement method, two different types of phantoms with known water-filled or water displacement volume were scanned (Additional file 2). The first type of phantoms consisted of differently shaped balloons filled with a known volume of water (50.0 mL, 10.0 mL, 7.0 mL) and non-ionic iodinated contrast medium (1.0 mL, 0.5 mL, 0.4 mL Xenetix® 300) positioned in a water-filled bucket. The balloons were allowed to touch the surface of the water. An abdominal CT-scan was performed and the obtained CT recordings were analysed by encircling the area of the phantom and the sum of slices with Amira software (Fig. 3).

**Fig. 4:**
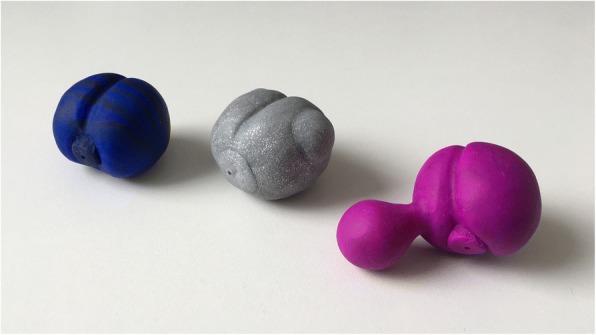
Three different shaped spheres. Regular, irregular and one with “a paraprostatic cyst” (from left to right)

**Fig. 5:**
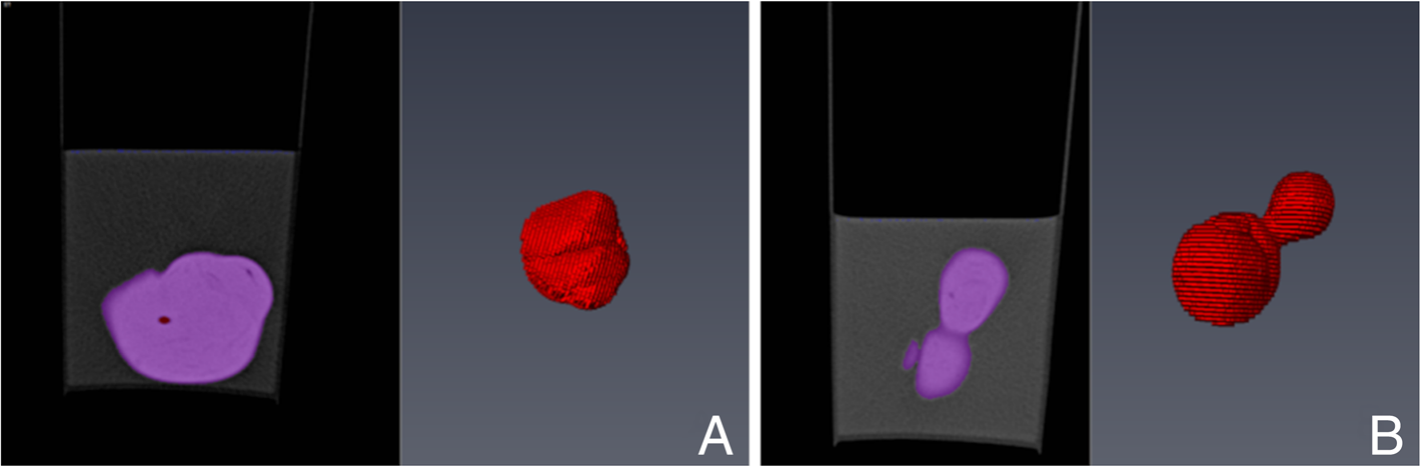
Segmentation of different shaped spheres through Amira. Irregular sphere (**a**) and sphere with “paraprostatic cyst” (**b**) after segmentation using Amira software

The second group consisted of three spheres (thermoset polyvinyl chloride modelling mass, STAEDTLER Mars GmbH & Co. KG, Nuremberg, Germany) shaped like a prostate (Figs. 4 and 5), one regular (ca. 4 × 4 × 3 cm^3^), one irregular (ca. 4 × 4.5 × 3 cm^3^) and one with a “paraprostatic cyst” (ca. 5.5 × 4 × 3.5 cm^3^) (Additional file 2).

The volume of the phantoms from this group was measured by water displacement, positioning the phantoms in a 250 cm^3^ measuring cup filled with 100 cm^3^ water. The volume was directly read off the measuring scale (5 cm^3^ steps), subtracting 100 cm^3^. The phantoms were scanned in a water-filled bucket (100 mL) and CT scans were analysed with Amira as described above.

Furthermore, five (four intact, one neutered) canine cadavers (5.1–35.2 kg, one-15 years) which had been euthanised due to other diseases at Small Animal Clinic, University of Veterinary Medicine Hannover, Foundation, underwent CT examination without applying contrast medium (Additional file 2). The dog owners’ consent had been previously obtained. After scanning, a necropsy was carried out; the prostate was removed and surrounding tissue was separated from the prostate gland. The prostate volume was determined by water displacement, positioning the prostate in a 250 cm^3^ measuring cup filled with 100 cm^3^ water. The volume was directly read off the measuring scale (5 cm^3^ steps), subtracting 100 cm^3^. Measurements were repeated twice and the mean value (+/− standard deviation) was determined.

### Statistical analysis

Statistical analysis was carried out with SAS® Enterprise Guide® 7.1 (Statistical Analysis Software, Heidelberg, Germany). Normal distribution was analysed with the Kolmogorov- Smirnov test or Shapiro-Wilk test. Differences of the mean values between the groups were tested with the Ryan-Einot-Gabriel-Welsch multiple range-test. *P*-values less than 0.05 were assumed to be statistically significant. Depending on normal distribution correlations between the age of the dog or length of the sixth lumbar vertebra, the respective prostate volumes were tested by the Pearson or Spearman correlation test. As Rv showed right skewed distribution, those values were logarithmised and further statistical analysis was performed (LogRv). Differences in volume of the prostates in regard to castration or prostate structure were examined with a two-way analysis of variance and post-hoc Tukey test. The evaluation of accuracy of the assessment method was performed by Bland-Altman plots with GraphPad Prism (Graphpad Software, Version 7, San Diego, USA 2003) and linear regression analysis by SAS.

## Results

### Phantom and cadaver measurements

**Fig. 6:**
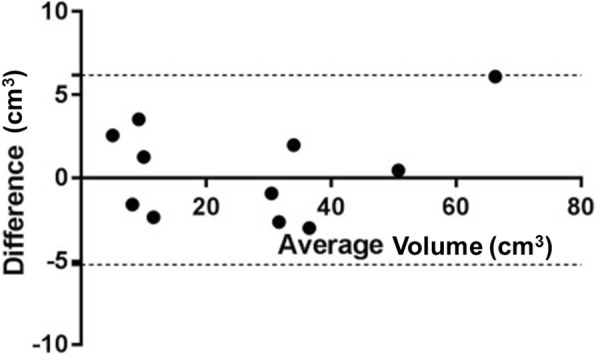
Bland-Altman-plot for evaluating accuracy of volumetric measurements. Interrupted lines represent upper and lower 95% limits of agreement

**Fig. 7:**
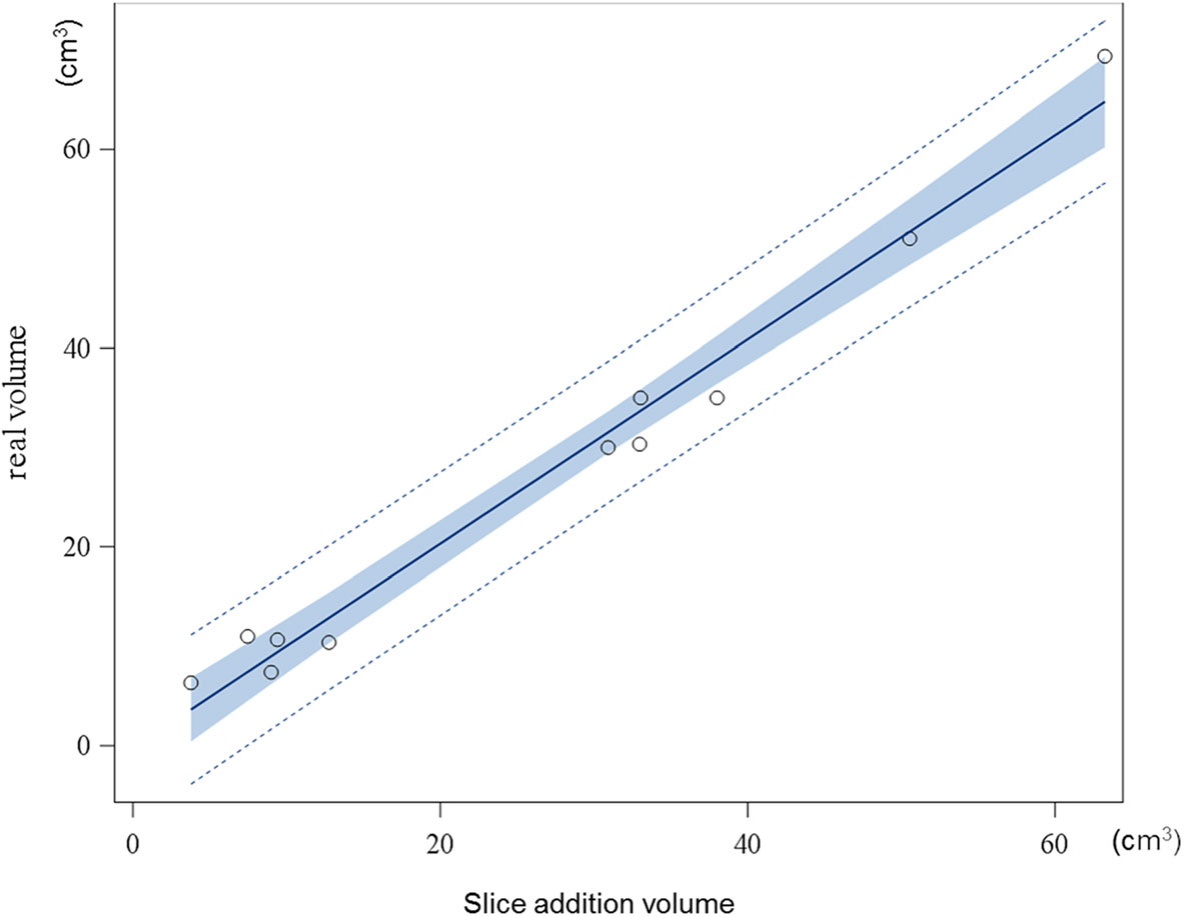
Linear regression analysis of real volume and phantoms/cadaver.Blue area represents 95% confidence limits, dotted lines represent 95% prediction limits

Comparing volumetric measurements in CT images with actual volumes measured by water displacement or phantoms with known volumes, the results showed a low bias (bias = 0.48 (+/− 2.90)) (Fig. 6). The ANOVA showed no significant differences in average volumes between the CT assessment method and actual volume (*p* = 0.766). Furthermore, linear regression exhibited a significant relation (*p* < 0.001, R-Square = 0.98) between volumetric measurements and actual prostate values (Fig. 7).

### Volumetric measurements

**Fig. 8:**
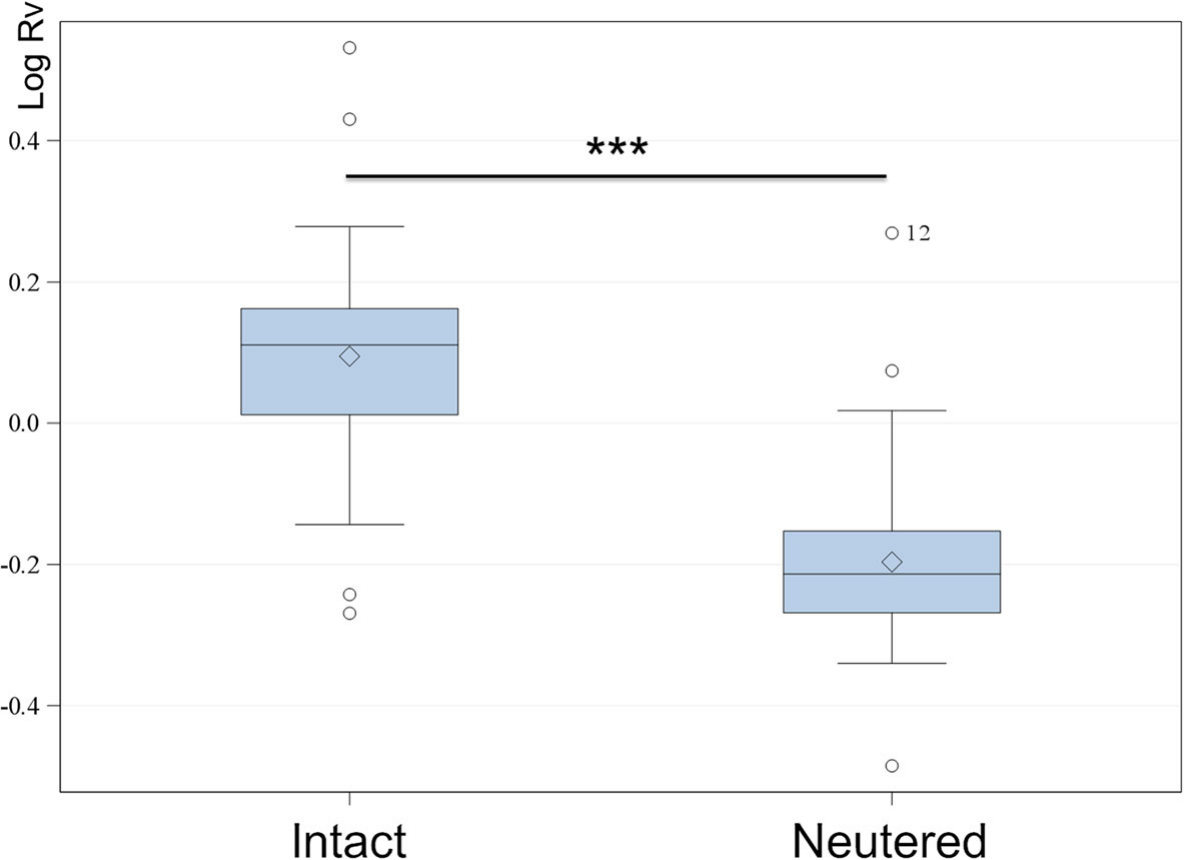
Differences in log ratio volume between intact and neutered dogs. *p* < 0.05 = *, *p* < 0.01 = **, *p* < 0.001 = ***, Rv = Ratio volume

The mean volume of the prostate gland was 58.6 cm^3^ (ranging from 0.6 to 1600.5 cm^3^). The mean ratio volume measured was 1.1 (ranging from 0.3 to 3.4). The ratio volume was statistically significantly affected by status of castration and prostate structure. The mean ratio volume was 1.3 in intact male dogs and 0.7 in neutered dogs. The prostate ratio volume of intact male dogs was significantly higher than that of neutered dogs (*p* < 0.001) (Fig. 8).

**Fig. 9:**
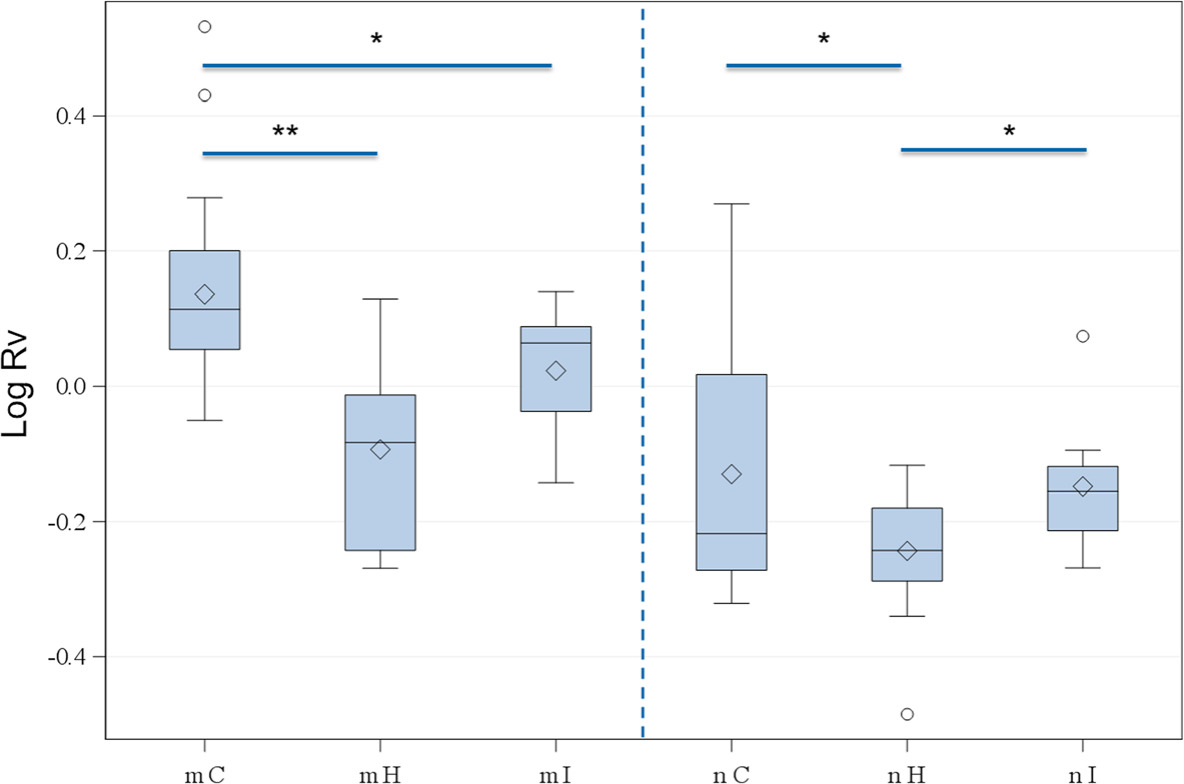
Differences in log ratio volume between structure groups for intact and neutered dogs. m = male intact, n = neutered, H = homogeneous, I = inhomogeneous, C = cystic; Rv = Ratio volume. *p* < 0.05 = *, *p* < 0.01 = **, *p* < 0.001 = ***

Homogeneous prostates showed the lowest mean ratio volume in intact (Rv = 0.9) and castrated dogs (Rv = 0.6), followed by inhomogeneous prostates (intact: Rv = 1.1 and neutered: Rv = 0.7, respectively). Canine prostates with cystic structures exhibited the highest mean volume values in intact and neutered dogs (i: Rv = 1.4 and n: Rv = 0.8, respectively). In intact dogs, differences in volume were significant between structure H and C (*p* = 0.001) as well as between structure I and C (*p* = 0.020). No statistically significant differences in volume were found between the structures H and I. In neutered dogs, the volume of prostates grouped as structure H and I as well as structure H and C differed statistically significantly (*p* = 0.015 and p = 0.015, respectively). No significant difference in prostate volume was found between structure I and C (Fig. 9).

### Volume regarding patient’s age and body size (length of sixth lumbar vertebra)

There were significant differences in volume between age groups in intact male dogs (F = 9.35; *p* < 0.001), but no significances were found in castrated male dogs (Table 3). Significant differences in volume were found between age groups 1 and 2 as well as between age groups 1 and 3 in intact male dogs (Table 3).

**Table 3:** Ryan-Einot-Gabriel-Welsch and Quiot (REGWQ) multiple range test

Intact Male Dogs				Castrated Male Dogs			
Age group	Mean Log Rv	N	REGWQ Grouping	Age group	Mean Log Rv	N	REGWQ Grouping
1	−0.03	12	A	1	−0.23	6	A
2	0.1	19	B	2	−0.18	11	A
3	0.15	27	B	3	−0.19	20	A

REGWQ for log ratio volume of intact (left) and castrated male dogs (right). Means with the same letter were not significantly different

*N* number of patients, *Rv* Ratio volume

The Pearson correlation showed a significant positive correlation between the ratio volume and age in intact male dogs (r = 0.52, *p* < 0.001), but not in neutered dogs (*p* = 0.407) (Table 4). Furthermore, there was a significant positive correlation between prostate volume and length of the sixth lumbar vertebra for both, intact (r = 0.63, *p* < 0.001) and castrated dogs (r = 0.48, *p* = 0.003). The correlation was higher in intact male dogs (Table 4).

**Table 4:** Pearson Correlation

Volume – length 6th lumbar vertebra		Ratio volume - age	
intact	neutered	intact	neutered
r = 0.63 *p* < 0.001	r = 0.48 *p* = 0.003	r = 0.52 *p* < 0.001	r = 0.14 *p* = 0.407

Correlation between prostatic volume and length of 6th lumbar vertebra and between ratio volume and age for intact and castrated dogs

*r* correlation coefficient

## Discussion

### Validation of accuracy of the slice addition technique

The CT is recommended as a beneficial tool for investigating the prostate gland of dogs [16, 19]. However, literature on volumetric measurements in CT is rare and validation of the used method is often missing.

Therefore, the primary objective in the present study was to validate the slice addition technique of Amira to measure prostate and phantom volumes. The comparison of these measurements with the actual volumes of the phantoms and cadaver prostates showed an excellent agreement.

These results are in accordance with the results of Moss et al. who used a slice addition technique in CT to measure the volume of the canine spleen, liver and kidney [22]. Thereby, accuracy was determined after euthanasia of the dogs and measuring actual organ volume by water displacement, comparable to the present study. They came to the conclusion that this procedure was accurate to +/− 5% of the organ volume measured by water displacement, but analysis of the prostate gland was missing [22]. In the present study, an even smaller divergency of +/− 0.8% was observed.

Other techniques like a volume rendering software tool to measure prostate volume in CT used in the studies of Lee et al. and Choi et al. are promising but the accuracy of this technique was not validated [17, 19].

Schulze et al. measured prostate volume in CT with a formula of an ellipsoid [20] although it is known that calculations with this kind of formula result in an over- or underestimation of the prostate volume [13, 14]. In addition, accuracy was not verified [22]. To the best of our knowledge, the present study is pioneering in comparing actual volume of the canine prostate gland with volumetric measurements in computed tomography.

### Prostate volume analysis

The mean prostate volume of all dogs evaluated in this study was 58.6 cm^3^. This value is higher in comparison to other studies (Table 5). In most studies, the examined dogs were young, healthy and the number of breeds was limited [10, 12, 19, 20, 23], while in the present study, the castration status, health status, breed, age and the size of the dogs differed. Schulze et al., Ruel et al. and Atalan et al. examined 50, 100 and 154 dogs, respectively, healthy male intact dogs of different breeds, ages and sizes and found average prostate sizes of 12–30 cm^3^ [10, 12, 20]. Prostate health status might be the reason for the differences in prostate volumes in these studies and the higher volume in the present study. Altered prostates showed significantly higher volume values compared to prostates with homogenous morphology in the present study. Since about 73% of the examined prostates showed morphological changes in CT examination in the present study, volumes of healthy prostates must be considered smaller. Research carried out by Choi et al. and Lowseth et al. focussed on eight and 15 healthy beagle dogs, respectively [19, 23]. The small breed and the prostate health status of these dogs led to a lower prostate volume compared to the present study. Another reason for the lower volume values of Schulze et al. [20], Ruel et al. [12], Atalan et al. [10] and Nair et al. [15] might be the determination of the volume using different formulas which over- or underestimate the actual prostate volume. Lower volumes can result from measurements of length, width and height that do not include a focal cyst which can be as large as the prostate itself [5]. Calculating a volume using these measurements can lead to an underestimation of the prostate volume.

**Table 5:** Mean prostatic volumes and standard deviation measured in different studies

Study	Total mean volume (cm^3^)	Mean volume intact dogs (cm^3^)	Mean volume neutered dogs (cm^3^)
Haverkamp et al.	58.6 (+/− 188.6)	92.4 (+/− 235.93)	5.7 (+/− 6.42)
Schulze et al. [20]	30.02 (+/− 27.52)	30.02 (+/− 27.52)	–
Nair et al. [15]	20.23 (+/− 1.34)	–	–
Ruel et al. [12]	18.9 (+/− 15.5)	18.9 (+/− 15.5)	–
Choi et al. [19]	18.56 (+/− 7.72)	–	–
Atalan et al. [10]	12.3	12.3	–
Lowseth et al. [23]	9.64 (+/− 2.31) – 22.0 (+/− 3.13)	9.64 (+/− 2.31) – 22.0 (+/− 3.13)	–

### Volume and status of castration

Analysis of prostate volume in castrated dogs is missing in past literature [16]. In the present study, castrated dogs showed significantly smaller prostate volumes compared to intact dogs. This finding corresponds to literature that found canine prostate atrophy as a result of androgen deprivation due to castration [24]. As BPH development was found to be androgen-dependent [25], there is less basis for development of prostatic disease in neutered dogs [6, 26]. Just one study investigated the prostate of intact and neutered dogs in diagnostic imaging. Cadavers were evaluated ultrasonographically [9]. However, volume measurement was performed in only two castrated dogs and a statistical analysis could not be carried out. In the present study, a group of 37 castrated dogs could be investigated in addition to intact male dogs.

### Volume in differently structured prostates

In addition to the status of castration, it could be shown that prostate structure has distinct effects on the prostate volume. All in all, volume increased from homogeneous to inhomogeneous to cystic-structured prostates. In intact dogs, homogeneous and inhomogeneous prostates showed statistically analogue volume values and differed significantly from cystic prostates. Similar results were observed in a study by Kuhnt et al. that compared measurements of length, height and width with the organ’ s texture [16]. The ratio of the length of cystic-structured prostates differed significantly from structures H and I, while the height was just different between groups H and C and width showed no significant differences. Since both studies used almost similar patient data, the measurements can be compared directly with each other. The volumetric measurements in the present study did show significant differences in volume according to the alteration in prostate structure. These results were comparable with the measurements of length in CT in the study by Kuhnt et al., while the measurements of width and height could not provide the same information [16]. Consequently, volumetric measurements were able to provide more information about differences due to varying prostate alterations. Significant differences in volume are missing between structures H and I in intact dogs. In other studies, inhomogeneous alterations of the canine prostate were found to be associated with BPH and prostatitis [2, 18]. Thus, missing differences may be a result of early stage BPH, in which parenchymal alterations are visible in CT, while the overall size of the organ is not yet increased.

Despite a lack of hormonal influence, there were some discrepancies in volumes of castrated dogs between the structure groups in this study. Prostatic volume values of groups I and C were statistically analogue in castrated dogs and differed significantly from group H. Accordingly, suspicious prostates could be easily distinguished from normal prostate glands by volumetric measurements. As there was no significant difference in volume between groups I and C, volumetric measurements seem to be unsuitable for differentiating between these pathological alterations of the prostatic gland in neutered dogs. This is in contrast to intact male dogs, where it is possible to differentiate prostatic alterations. Thus, alterations of the gland in neutered dogs appear rather mild compared to intact dogs.

### Prostate volume in relation to body size and age

Prostate volume was well correlated with age as well as body size. The length of the sixth lumbar vertebra has been used to predict body size in literature before [16, 17, 19]. However, to the best of our knowledge, the relationship of the sixth lumbar vertebra length to the prostate volume has not been previously proven. In the present study, we could establish a coherence of length of the sixth lumbar vertebra to the prostate volume. With increasing body size, the prostate volume increased in both, male intact and castrated dogs. Nonetheless, the correlation was higher within intact dogs. Choi et al. used the length of the sixth lumbar vertebra to predict limits of the normal range of prostate length, height and width in CT. However, the prostate volume was not analysed in relation to the sixth lumbar vertebra [19]. Studies by Atalan et al. used the bodyweight [9, 10] and Ruel et al. used the body height, weight, length of left kidney and aortic diameter to predict body size [12]. All these parameters were significantly correlated with prostate volume measured in ultrasound. No previous study investigated the relationship of body size and prostate volume in neutered dogs. In our study, a positive correlation could be discovered for neutered dogs. Castration and following prostatic atrophy might be responsible for the lower correlation in castrated dogs compared to intact dogs.

There was also a good positive correlation between age and prostate volume in male intact dogs, while no significant relation was found in neutered dogs. These findings are in agreement with other studies [10, 12, 15, 20]. Atalan et al. showed a significant correlation between age and prostate volume calculated by ultrasound measurements (correlation coefficient = 0.581) [10]. In a study by Ruel et al., prostates of 100 healthy intact male dogs were analysed ultrasonographically and the volume was determined by a formula [12]. There was a weak but significant correlation between age and volume (correlation coefficient = 0.27). Furthermore, there was also a positive correlation for the prostate length and width with age, but no correlation was found between age and height. This shows that volumetric measurements are more suitable for comparison to single parameter measurements to describe prostate dimensions and might represent a beneficial alternative for evaluating prostate size in future. However, no data were published for neutered dogs. In our study, castrated dogs showed no correlation between prostate volume and age. We consider the status of castration as a reason for the absence of correlation in these dogs.

### Limitations

The present study focused on volumetric measurements of the prostate gland in CT images. Consequently, the confirmation of prostatic disorders by cytology or histology was neglected. Concerning neutered dogs, the time between castration and CT-evaluation might have an effect on findings of prostate morphology and volume but was unknown in this study. Since the reviewer who measured volume by Amira allocated patients to different groups, the reviewer was not blind to the patient’s age, body size, castration status and prostate architecture during volume analysis. Thus, this could be a possible source of bias in the volume analysis. As only one radiologist read the CT images to avoid inter-observer variability, this limits the assessment of practicality of this procedure in clinical routine.

## Conclusion

The slice addition technique carried out in Amira represents a precise way to analyse the volume of the canine prostate gland in CT. Volumetric measurements of the canine prostate gland in CT images provide information about the prostate structure, castration status, age and body size of the patient. Therefore, prostate volume is a relevant parameter for evaluating prostate health status. These measurements might represent a promising alternative to measurements of length, height or width to describe prostate size. The length of the sixth lumbar vertebra can be used to relate prostate volume to the body size. Further studies should focus on the validation of volume measurement techniques that can be carried out more rapidly to apply volumetric measurements to clinical routine.

## Additional files

Additional file 1: Patient data and volumetric measurements m = male intact; n = male neutered; H = homogeneous; I = inhomogeneous; C = cystic; − = no data available. (XLS 43 kb)

Additional file 2: Phantom and cadaver measurements PVC = polyvinyl chloride; m = male intact, n = male neutered; H = homogeneous; I = inhomogeneous; C = cystic; − = no data available. (XLS 26 kb)

## Abbreviations

BPH: Benign prostatic hyperplasia
C: Cystic
CT: Computed tomography
D: Depth
DL: Depth longitudinal
DT: Depth transversal
H: Homogeneous
He: Height
I: Inhomogeneous
L: Length
N: Number
R: Rectum
REGWQ: Ryan-Einot-Gabriel-Welsch and Quiot
ROI: Range of interest
Rv: Ratio volume
V: Volume
W: Width
